# Heterozygous Insulin Receptor *(INSR)* Mutation Associated with Neonatal Hyperinsulinemic Hypoglycaemia and Familial Diabetes Mellitus: Case Series

**DOI:** 10.4274/jcrpe.galenos.2019.2019.0106

**Published:** 2020-11-25

**Authors:** Aashish Sethi, Nicola Foulds, Sarah Ehtisham, Syed Haris Ahmed, Jayne Houghton, Kevin Colclough, Mohammed Didi, Sarah E. Flanagan, Senthil Senniappan

**Affiliations:** 1 Alder Hey Children’s Hospital, Department of Paediatric Endocrinology, Liverpool, UK; 2 Wessex Clinical Genetics Services, Clinical Genetics, Southampton, UK; 3 Mediclinic City Hospital, Deparment of Paediatric Endocrinology, Dubai, UAE; 4 Countess of Chester Hospital, Department of Endocrinology, Chester, UK; 5 Royal Devon and Exeter NHS Foundation Trust, Department of Molecular Genetics, Exeter, UK; 6 University of Exeter Medical School, Institute of Biomedical and Clinical Science, Exeter, UK

**Keywords:** INSR mutation, congenital hyperinsulinism, neonatal hyperinsulinemic hypoglycemia, familial diabetes mellitus

## Abstract

Mutations in the *insulin receptor* (*INSR*) gene are associated with insulin resistance and hyperglycaemia. Various autosomal dominant heterozygous *INSR* mutations leading to hyperinsulinemic hypoglycaemia (HH) have been described in adults and children (more than 3 years of age) but not in the neonatal period. Family 1: A small for gestational age (SGA) child born to a mother with gestational diabetes presented with persistent hypoglycaemia, was diagnosed with HH and responded well to diazoxide treatment. Diazoxide was gradually weaned and discontinued by 8 months of age. Later, the younger sibling had a similar course of illness. On genetic analysis a heterozygous *INSR* missense variant p.(Met1180Lys) was found in the siblings, mother and grandfather but not in the father. Family 2: A twin preterm and SGA baby presented with persistent hypoglycaemia, which was confirmed as HH. He responded to diazoxide, which was subsequently discontinued by 10 weeks of life. Genetic analysis revealed a novel heterozygous *INSR* missense variant p.(Arg1119Gln) in the affected twin and the mother. Family 3: An SGA child presented with diazoxide responsive HH. Diazoxide was gradually weaned and discontinued by 9 weeks of age. Genetic analysis revealed a novel heterozygous *INSR* p.(Arg1191Gln) variant in the proband and her father. We report, for the first time, an association of *INSR* mutation with neonatal HH responsive to diazoxide therapy that resolved subsequently. Our case series emphasizes the need for genetic analysis and long-term follow up of these patients.

**What is already known on this topic?**

Homozygous or compound heterozygous mutations in the *insulin receptor (INSR)* gene, linked with Rabson-Mendenhall and Donohue syndromes, have been described to cause neonatal hypoglycaemia.

**What this study adds?**

We report the first case-series of neonatal hyperinsulinemic hypoglycaemia associated with heterozygous mutations in *INSR* leading to variable phenotype among the family members, ranging from neonatal hypoglycaemia to adult-onset diabetes mellitus. This highlights the importance of genetic analysis and long-term follow up of these patients.

## Introduction

*Insulin receptor (INSR)* is a trans-membrane receptor from the tyrosine kinase family where insulin binds to two distinct sites on each subunit of the receptor (1,2). *INSR *mutations are associated with severe insulin resistance (IR) phenotypes, such as Rabson-Mendenhall, Donohue and type A IR syndrome (3,4). Rabson-Mendenhall and Donohue syndromes are recessively inherited conditions leading to decreased expression of receptor, blockage of receptor transport to the plasma membrane or decreased insulin binding, associated with extreme IR. Type A IR is associated with mono allelic *INSR* missense mutations, which cause a less severe phenotype and are most likely to occur within the tyrosine kinase domain (4).

Insulin is an anabolic peptide hormone secreted by the beta cells of pancreatic islets. Inappropriate secretion of insulin leads to persistent/recurrent hypoglycaemia (hyperinsulinemic hypoglycaemia, HH), which can result from a mutation in a number of different genes (5,6).

There are several reports of autosomal dominant heterozygous *INSR* mutations causing hypoglycaemia in adults but to the best of our knowledge there is no data about heterozygous *INSR* mutations causing neonatal HH (7,8,9,10,11,12).

In this study, we report three different families presenting with variable clinical manifestations, ranging from neonatal HH to adult onset type 2 diabetes mellitus, associated with heterozygous *INSR* mutation.

## Case Reports

### Family 1

A 13-day-old female baby (proband) was referred to the tertiary endocrine unit with persistent neonatal hypoglycaemia since three hours of life (capillary blood glucose: 1.9 mmol/L). She was born by emergency caesarean section due to breech presentation at 37 weeks gestation, weighing 2.41 kg [-2.42 standard deviation score (SDS)] and measuring 47 cm in length (-1.15 SDS). There was a history of gestational diabetes in the mother requiring insulin treatment. The hypoglycaemia was initially managed with high concentration dextrose containing fluids [glucose infusion rate (GIR): 18.1 mg/kg/min] and intravenous glucagon (10 mcg/kg/hr) infusion. HH was confirmed on hypoglycaemia screen (Table 1). The echocardiogram was normal and she was subsequently commenced on oral diazoxide at 5 mg/kg/day and chlorothiazide at 7 mg/kg/day. She responded well to diazoxide which enabled weaning off the intravenous fluids and intravenous glucagon. She was fully established on oral feeds prior to discharge. During subsequent follow up, the diazoxide and chlorothiazide were slowly weaned and fully discontinued by eight months of age following which a 16-hour controlled fast showed complete resolution of HH (Table 2).

The second child was born at 38 week of gestation by elective caesarean, weighing 2.43 kg (-2.35 SDS) and measuring 48 cm in length (-0.62 SDS). There was no history of birth asphyxia. The mother once again developed gestational diabetes but did not require insulin treatment unlike the previous pregnancy. At 10 hours of age she had symptomatic hypoglycaemia (capillary blood glucose: 1.9 mmol/L) and was managed with intravenous dextrose containing fluids (GIR of 11.5 mg/kg/min). At 48 hours of age, a hypoglycaemia screen (Table 1) was undertaken which confirmed HH. Echocardiogram did not reveal any underlying cardiac abnormality. Diazoxide was started at 3 mg/kg/day to achieve normoglycaemia. Oral chlorothiazide (7 mg/kg/day) was added in conjunction with diazoxide. The intravenous fluids and glucagon were gradually weaned and the patient was established on full enteral feeds prior to discharge. During subsequent follow up, the diazoxide and chlorothiazide were slowly weaned and fully discontinued by 11 months of age, following which she underwent a controlled fast appropriate for age which confirmed the resolution of HH (Table 2).

The proband’s mother had gestational diabetes mellitus during both pregnancies, requiring insulin treatment during the first pregnancy. She had normal body mass index (BMI) (BMI 24 kg/m^2^) and had no features of IR, such as acanthosis nigricans (AN), previous menstrual abnormalities or hirsutism (HR). The proband’s maternal grandfather (BMI 25 kg/m^2^) was diagnosed with type 2 diabetes mellitus at the age of 45 years and was treated with metformin. On examination he did not have any signs of IR or any history of hypoglycaemia.

**Figure 1:**
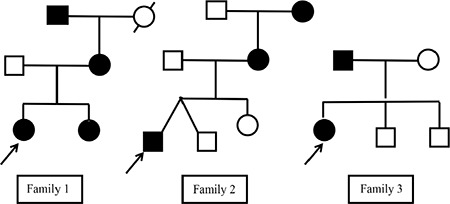
Pedigree chart showing autosomal dominant inheritance in the families; circle denotes females, square males. The proband is indicated by arrow mark in each family, dotted symbol denote person with diabetes with unknown mutation status and solid symbols denote affected subjects with (Family 1) *INSR* gene p.(Met1180Lys) or (Family 2) *INSR* gene p.(Arg1119Gln) or (Family 3) *INSR* gene c.3572G>A, p.(Arg1191Gln) mutation

Targeted next generation sequencing of the known hyperinsulinism genes identified a novel heterozygous *INSR* variant p.(Met1180Lys) (c.3539T>A) in the proband, her sister, mother and maternal grandfather. No further disease-causing variants were identified (Figure 1).

### Family 2

The proband was a preterm (36 weeks gestation) twin, born to non-consanguineous parents by elective LSCS with a birth weight of 2.025 kg (-3.75 SDS). Hypoglycaemia was recorded on the first day of life (capillary blood glucose 1.1 mmol/L). There was no history of birth asphyxia. The second twin had a birth weight of 2.56 kg (-1.95 SDS) and did not have any hypoglycaemia during the neonatal period. There was a maternal history of hypothyroidism, which was well controlled on thyroxine. The hypoglycaemia was initially managed with high concentration dextrose containing fluids (GIR: 15.2 mg/kg/min) and intravenous glucagon (10 mcg/kg/hr) infusion. The hypoglycaemia screen (Table 1) confirmed HH. Echocardiography revealed moderate pulmonary stenosis and small patent foramen ovale. The child was subsequently commenced on diazoxide at 7.5 mg/kg/day and spironolactone at 2 mg/kg/day. He responded well to diazoxide which enabled weaning off the intravenous fluids and intravenous glucagon. He was fully established on formula feeds prior to discharge. During subsequent follow up, the diazoxide and spironolactone were slowly weaned and fully discontinued by 10 weeks of age.

The proband’s elder sibling is a 3-year-old healthy girl with no history of neonatal hypoglycaemia. The proband’s mother did not have any symptoms related to hypoglycaemia and was able to fast for prolonged hours during Ramadan. She had a normal BMI, no signs of IR, such as AN, and there was no previous history of menstrual abnormalities or HR. There was a history of type 2 diabetes in the maternal grandfather and maternal great grandmother.

Targeted next generation sequencing of the known hyperinsulinism genes identified a novel heterozygous *INSR* variant p.(Arg1119Gln) (c.3356G>A) in the proband and her mother. No further disease-causing variants were identified. Samples from the twin and older sister were not available for testing (Figure 1).

### Family 3

The proband was born was born at 37 week of gestation, weighing 2.20 kg (-1.51 SDS). There was no history of birth asphyxia. At a few hours of age she developed symptomatic hypoglycaemia (capillary blood glucose: 1.1 mmol/L) and was managed with intravenous dextrose containing fluids. At 48 hours of age, a hypoglycaemia screen (Table 1) was undertaken which confirmed HH. Echocardiogram did not reveal any underlying cardiac abnormality. Diazoxide (5 mg/kg/day) in conjunction with chlorothiazide (7 mg/kg/day) was started. The intravenous fluids and glucagon were gradually weaned and the patient was established on full enteral feeds prior to discharge. During subsequent follow up, the diazoxide and chlorothiazide were slowly weaned and fully discontinued by nine weeks of age.

The proband’s father had a normal BMI and did not have any symptoms related to IR, such as AN, or glucose variability (HbA1c: 36 mmol/L, fasting glucose: 4.6 mmol/l). The proband has two elder siblings who did not have any hypoglycaemia or symptoms related to IR.

Targeted next generation sequencing of the known hyperinsulinism genes identified a novel heterozygous *INSR* variant p.(Arg1191Gln) (c.3572G>A) in the proband and her father. No further disease-causing variants were identified (Figure 1).

## Discussion

The human *INSR* is a heterotetramer composed of two a and two β subunits. The α subunit is entirely extracellular and the β subunit has extracellular, transcellular and intracellular domains that expresses tyrosine kinase activity. Insulin binds to α subunits and stimulates β subunit auto phosphorylation and kinase activity (1,2). A single gene, *INSR* located at 19p13.2 of 22 exon length, codes for both α and β subunits (13). Homozygous and compound heterozygous mutations in *INSR* lead to severe IR (Donohue syndrome, Rabson-Mendenhall syndrome), whereas the heterozygous mutations in *INSR* cause the milder phenotype of IR syndrome (14,15).

Heterozygous mutations in *INSR* cause IR in coexistence with hypoglycaemia, which may be due to selective impairment of *INSR* function in skeletal muscle causing defective peripheral glycogen formation and IR whilst the preserved *INSR* function in the liver leads to suppressed hepatic glucose production causing hypoglycaemia (10,16). This is possibly due to the differential effect of insulin on the phosphorylation of the *INSR* substrates (IRS) -1 and -2 in the skeletal muscle and liver (16). IRS-2 is constitutively phosphorylated due to the increased binding of the kinase regulatory loop binding domain of IRS-2 to the mutated receptor, preventing further activation by insulin or insulin-like growth factor (IGF) 1 in the muscles, whereas IRS-1 phosphorylation is normally activated by basal as well as stimulated levels of insulin (17). This would explain the relative IR in the skeletal muscle in contrast to the insulin sensitivity in the liver.

Only a few patients with a heterozygous *INSR* mutation associated with episodes of hypoglycaemia have been reported (Table 3), mainly demonstrating post-prandial hypoglycaemia following oral glucose tolerance test (8,9). Symptomatic fasting hypoglycaemia has been reported in a few patients; however, age appropriate controlled fasts were not undertaken (8,9,10,11). Symptomatic hypoglycaemia is reported to have occurred as young as three years of age in one patient, however HH was not confirmed (7). The signs of IR were noted in most of these patients while there is only minimal information provided about the individual family members. Enkhtuvshin et al (18) reported a heterozygous *INSR* mutation associated with type 2 diabetes mellitus in a mother and transient hypoglycaemia in both children at birth with the same mutation, however hyperinsulinemia was not documented and hypoglycaemia resolved within 10 hours in both the siblings.

There have been several reported cases associated with homozygous or compound heterozygous mutations causing Donohue syndrome (complete absence of functional insulin receptors) and leading to fasting hypoglycaemia which has been suggested as the effect of insulin on type 1 IGF receptors (19). However type 1 IGF receptors disappear from the liver in adult life (20), which doesn’t explain the several reported cases of hypoglycaemia in adult life associated with heterozygous *INSR* mutations (Table 3).

Neonatal HH has not been previously reported in association with heterozygous *INSR* mutations (7,8,9,10,11). The precise mechanism by which these mutations lead to diazoxide responsive neonatal HH, which resolves in infancy, is not clear. One of the possible explanations could be the effect of high levels of insulin on type 1 IGF receptors in the neonatal period with subsequent resolution of hypoglycaemia due to β-cell exhaustion and potential developmental changes in the expression of IGF1 receptor (21,22). In adults, the deposition of amyloid in the pancreas has been observed following chronic IR, which subsequently leads to type 2 diabetes mellitus (23).

In our study, among all three families, affected children were small for gestational age similar to the previously reported patients (9,18). Since insulin has a central role in controlling foetal growth, genetic factors, which impair insulin secretion or action, would be expected to reduce foetal growth. This has been demonstrated experimentally in transgenic mice lacking key intermediates of the insulin-signalling pathway (24). In our study, the affected adult family members were constitutionally lean, which suggests that peripheral IR might not only prevent glycogen storage in muscles, but also reduce lipogenesis and increase lipolysis in fat cells (9).

Although signs of IR, such as AN, and symptoms of androgen excess, such as menstrual abnormalities and hirsutism, are common in patients with *INSR* mutation, the families we describe did not have any such signs or symptoms, supporting the notion that different mutations in *INSR* lead to different phenotypes. Unfortunately we did not have access to insulin level data in the adult members of the families. Moreover, a common heterozygous mutation, p.Arg1174Gln, has been described to be associated with variable clinical phenotypes within the members of the same family (7).

Apart from the genetic background, the phenotype could also be influenced by environmental factors. Patients with IR and compensatory increase in insulin secretion may develop diabetes mellitus in later life when the ability to secrete insulin declines. This leads to postprandial glucose intolerance, followed by fasting hyperglycaemia and diabetes mellitus, which might be prevented by early inventions that include dietary and behavioural modifications. These observations suggest that patients with *INSR* mutation need to be followed up long term for other manifestations in later life.

Diazoxide is the first line treatment for congenital hyperinsulinism, which works as an agonist at KATP channel leading to termination of Ca+ dependent insulin release (25), however there is no data regarding the use of diazoxide in individuals with an *INSR* mutation causing neonatal HH. *INSR* mutations lead to high insulin levels due to IR and metformin has been used effectively in the adult population (10,11) although the mechanism is not clear. Avoidance of high glycaemic index foods may improve symptoms in the patients with post-prandial hypoglycaemia (11).

## Conclusion

We report the first series of heterozygous *INSR* gene mutations causing neonatal HH. This study also highlights that the same gene mutation can lead to variable phenotype within the same family members. Hence, a detailed genetic testing in the family is essential for long-term follow up. All our patients followed a relatively benign clinical course during infancy and the hyperinsulinism resolved in the first year of life, but further monitoring will inform us of the clinical course through to adult life. There is no consensus about the treatment of children with *INSR* mutations due to its rarity, but lifestyle modification could play a key role in long-term management.

## Figures and Tables

**Table 1:**
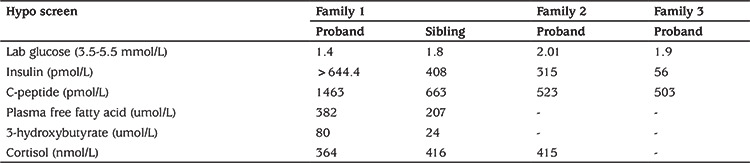
Biochemical parameters during the hypoglycemic episode

**Table 2:**
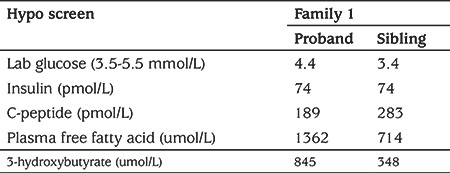
Biochemical evaluation at the end of 16 hour controlled fast

**Table 3:**
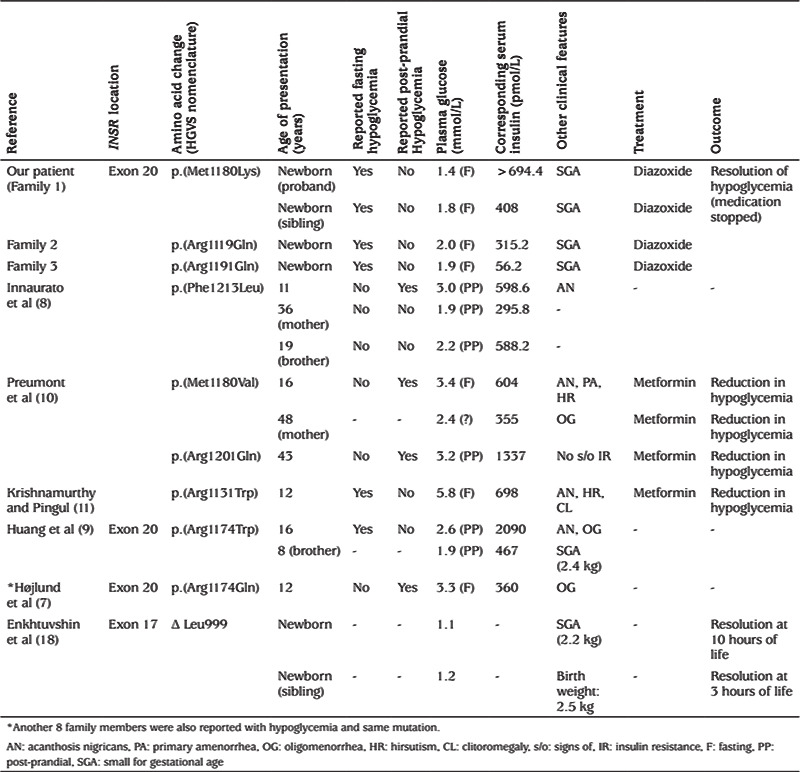
List of reported patients with hypoglycemia associated with heterozygous *INSR* mutation

## References

[1] Ebina Y, Ellis L, Yarnagin K, Edery M, Graf L, Clauser E, Ou JH, Masiarz F, Kan YW, Goldfine ID, Roth RA, Rutter WJ (1985). The human insulin receptor cDNA: the structural basis for hormone-activated transmembrane signaling. Cell.

[2] Ullrich A, Bell JR, Chen EY, Herrera R, Petruzzelli LM, Dull TJ, Gray A, Coussens L, Liao YC, Tsubokawa M, Mason A, Seeburg PH, Grunfeld C, Rosen OM, Ramachandran J (1985). Human insulin receptor and its relationship to the tyrosine kinase family of oncogenes. Nature.

[3] Taylor SI (1992). Lilly Lecture: Molecular mechanisms of insulin resistance. Lessons from patients with mutations in the insulin receptor gene. Diabetes.

[4] Longo N, Wang Y, Smith SA, Sharon D, DiMeglio LA, Giannella-Neto D (2002). Genotype-phenotype correlation in inherited severe insulin resistance. Hum Mol Genet.

[5] Galcheva S, Demirbilek H, Al-Khawaga S, Hussain K (2019). The Genetic and Molecular Mechanisms of Congenital Hyperinsulinism. Front Endocrinol (Lausanne).

[6] Senniappan S, Shanti B, James C, Hussain K (2012). Hyperinsulinaemic hypoglycaemia: Genetic mechanisms, diagnosis and management. J Inherit Metab Dis.

[7] Højlund K, Hansen T, Lajer M, Henriksen JE, Levin K, Lindholm J, Pedersen O, Beck-Nielsen H (2004). A novel syndrome of autosomal-dominant hyperinsulinemic hypoglycemia linked to a mutation in the human insulin receptor gene. Diabetes.

[8] Innaurato S, Brierley GV, Grasso V, Massimi A, Gaudino R, Sileno S, Bernardini S, Semple R, Barbetti F (2018). Severe insulin resistance in disguise: A familial case of reactive hypoglycemia associated with a novel heterozygous INSR mutation. Pediatr Diabetes.

[9] Huang Z, Li Y, Tang T, Xu W, Liao Z, Yao B, Hu G, Weng J (2009). Hyperinsulinaemic hypoglycaemia associated with a heterozygous missense mutation of R1174W in the insulin receptor (IR) gene. Clin Endocrinol (Oxf).

[10] Preumont V, Feincoeur C, Lascols O, Courtillot C, Touraine P, Maiter D, Vigouroux C (2017). Hypoglycaemia revealing heterozygous insulin receptor mutations. Diabetes Metab.

[11] Krishnamurthy M, Pingul MM (2016). A novel insulin receptor mutation in an adolescent with acanthosis nigricans and hyperandrogenism. J Pediatr Endocrinol Metab.

[12] Ardon O, Procter M, Tvrdik T, Longo N, Mao R (2014). Sequencing analysis of insulin receptor defects and detection of two novel mutations in INSR gene. Mol Genet Metab Rep.

[13] Seino S, Seino M, Nishi S, Bell GI (1989). Structure of the insulin receptor gene and characterization of its promoter. Proc Natl Acad Sci USA.

[14] Semple RK, Savage DB, O’Rahilly S (2011.). Genetics of severe insulin resistance. Oxford Textbook of Endocrinology and Diabetes. 2nd edition. Wass JAH, Stewart PM, Amiel SA, Davies MJ (eds). Oxford University Press.

[15] Thorton PS, Satin-Smith MS, Herold K, Glaser B, Chiu KC, Nestorowicz A, Permutt MA, Baker L, Stanley CA (1998). Familial hyperinsulinism with apparent autosomal dominant inheritance: clinical and genetic differences from autosomal recessive variant. J Pediatrics.

[16] Caruso M, Miele C, Oliva A, Condorelli G, Oriente F, Riccardi G, Capaldo B, Fiory F, Accili D, Formisano P, Beguinot F (2000). The IR1152 mutant insulin receptor selectively impairs insulin action in skeletal muscle but not in liver. Diabetes.

[17] Miele C, Caruso M, Calleja V, Auricchio R, Oriente F, Formisano P, Condorelli G, Cafieri A, Sawka-Verhelle D, Van Obberghen E, Beguinot F (1998). Differential role of insulin receptor substrate (IRS)-1 and IRS-2 in L6 skeletal muscle cells expressing the Arg1152 →Gln insulin receptor. J Biol Chem.

[18] Enkhtuvshin B, Nagashima S, Saito N, Wakabayashi T, Ando A, Takahashi M, Sakai K, Yamamuro D, Nagasaka S, Tamemoto H, Ishibashi S (2015). Successful pregnancy outcomes in a patient with type A insulin resistance syndrome. Diabetic Medicine.

[19] Ogilvy-Stuart AL, Soos MA, Hands SJ, Anthony MY, Dunger DB, O’Rahilly S (2001). Hypoglycemia and resistance to ketoacidosis in a subject without functional insulin receptors. J Clin Endocrinol Metab.

[20] Kasuga M, Van Obberghen E, Nissley SP, Rechler MM (1981). Demonstration of two subtypes of insulin-like growth factor receptors by affinity crosslinking. J Biol Chem.

[21] O’Brien TD, Rizza RA, Carney JA, Butler PC (1994). 1994 Islet amyloidosis in a patient with chronic massive insulin resistance due to antiinsulin receptor antibodies. J Clin Endocrinol Metab.

[22] Ogilvey-stuart AL, Soos MA, Hands SJ, Anthony MY, Dunger DB, O’Rahilly S (2001). Hypoglycemia and resistance to ketoacidosis in a subject without functional insulin receptors. J Clin Endocrinol Metab.

[23] Hull RL, Westermark GT, Westermark P, Kahn SE (2004). Islet amyloid: a critical entity in the pathogenesis of type 2 diabetes. J Clin Endocrinol Metab.

[24] Tamemoto H, Kadowaki T, Tobe K, Yagi T, Sakura H, Hayakawa T, Terauchi Y, Ueki K, Kaburagi Y, Satoh S, Sekihara H, Yoshioka S, Horikoshi H, Furuta Y, Ikawa Y, Kasuga M, Yazaki Y, Aizawa S (1994). Insulin resistance and growth retardation in mice lacking insulin receptor substrate-1. Nature.

[25] Kane C, Lidley KJ, Johnson PR, James RF, Milla PJ, Aynsley-Green A, Dunne MJ (1997). Therapy for persistent hyperinsulinemic hypoglycemia of infancy: understanding the responsiveness of β cells to diazoxide and somatostatin. J Clin Invest.

